# Magnesium aluminium zinc gallium, Mg_61.81_Al_12.77_Zn_61.41_Ga_24_

**DOI:** 10.1107/S2414314625007709

**Published:** 2025-09-05

**Authors:** Jingchao Yu, Changzeng Fan, Zhefeng Xu, Bin Wen, Lifeng Zhang

**Affiliations:** a State Key Laboratory of Metastable Materials Science and Technology Yanshan University,Qinhuangdao 066004 People’s Republic of China; b Hebei Key Lab for Optimizing Metal Product Technology and Performance Yanshan University,Qinhuangdao 066004 People’s Republic of China; c School of Mechanical and Materials Engineering North China University of Technology,Beijing People’s Republic of China; Benemérita Universidad Autónoma de Puebla, México

**Keywords:** crystal structure, high-pressure sinter­ing, inter­metallic, Mg_61.81_Al_12.77_Zn_61.41_Ga_24_

## Abstract

The title single-crystal was obtained during the synthesis of a Mg–Al–Zn–Ga alloy, which was achieved by subjecting the alloy to elevated pressures and temperatures. The compound crystallizes in the space group *Im*3 (No. 204), with seven distinct metal-atom sites. One of these is occupied by gallium, two by aluminium and zinc, one by zinc and magnesium, and three by magnesium. The structure model contains a vacancy-centred Bergman cluster and a 26-face polyhedron centred on one of the magnesium sites. The crystal structure framework in this study shows a marked similarity to previously examined frameworks, but also evinces significant differences [Edagawa *et al.* (1992). *Philos. Mag. B***65**, 1011–1023].

## Abstract 2

A new intermetallic compound with cubic symmetry belonging to the Mg–Al–Zn–Ga quaternary system is reported,.

## Graphical abstract

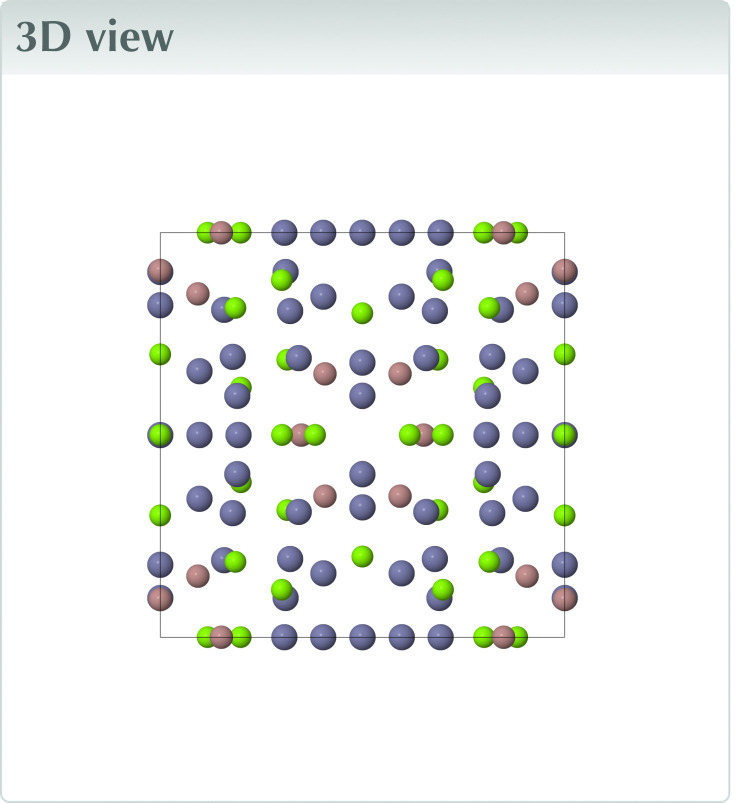

## Structure description

The Mg–Al–Zn–Ga system can serve as a lead-free brazing material, thus it has been extensively investigated. A quasi-crystalline phase Mg_39.5_Al_4.1_Zn_40.0_Ga_16.4_ has been reported in the Mg–Zn–Al–Ga system. The calorimetric and X-ray diffraction studies suggest that the quasiperiodic phase undergoes an exothermic transformation to an approximate crystalline phase (*a* = 36.93±0.06, *b* = 22.83±0.04, and *c* = 22.96±0.04 Å) at 630 K on heating at a rate of 20 K min^−1^ (Edagawa *et al.*, 1992 ▸). In another study, the icosa­hedral phase Mg_39.5_Al_14.35_Zn_40.0_Ga_6.5_ transforms to a 1/1 cubic approximant phase with *a* = 14.21 Å at 653 K (Edagawa *et al.*, 1993 ▸). It has been established that the two quasicrystalline approximant phases possess identical coordination polyhedra, yet divergent cell parameters.

**Figure 1:**
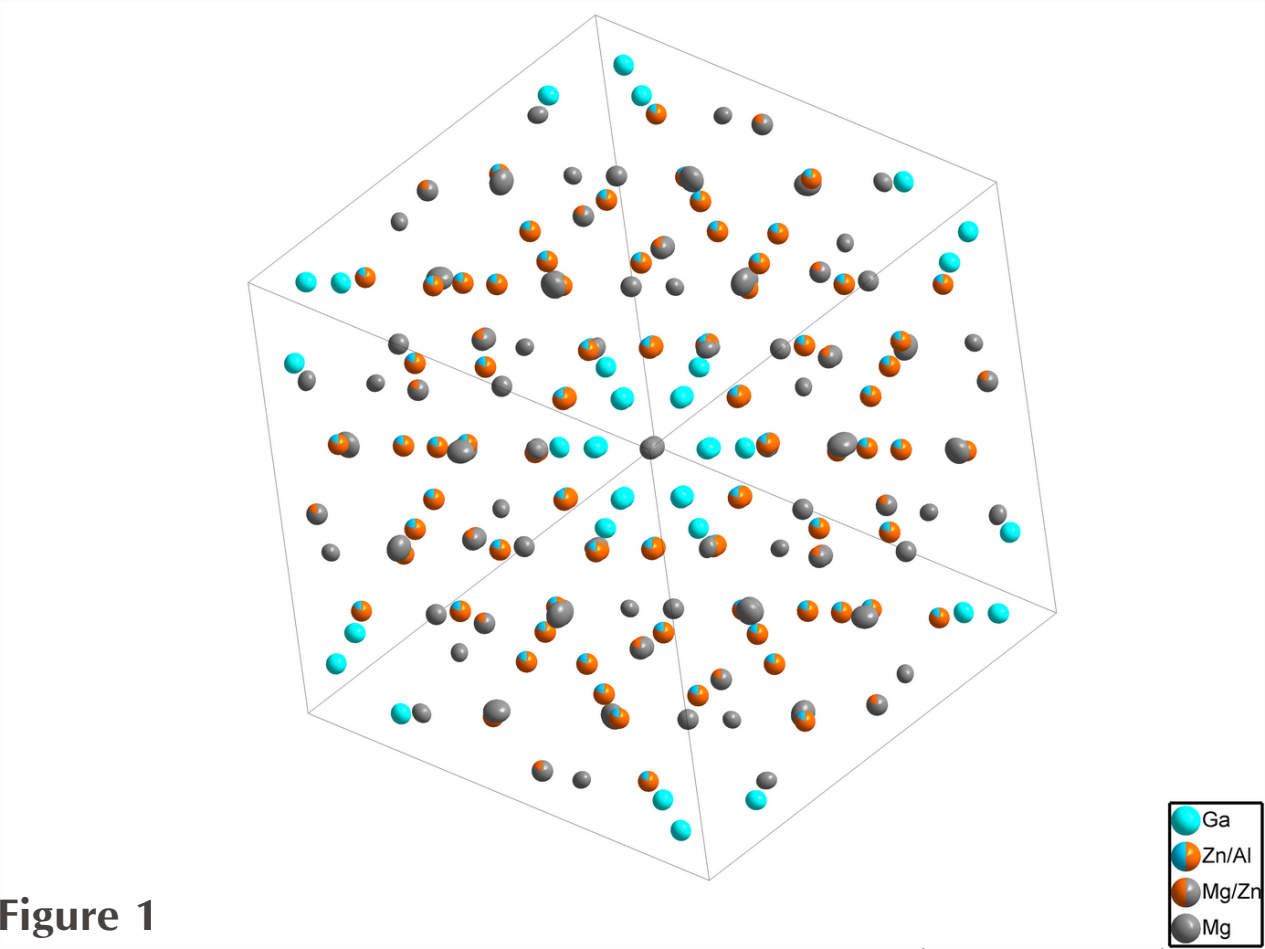
The crystal structure of Mg_61.81_Al_12.77_Zn_61.41_Ga_24_ (one unit cell) in a projection along the body diagonal, with displacement ellipsoids drawn at the 99% probability level.

**Figure 2:**
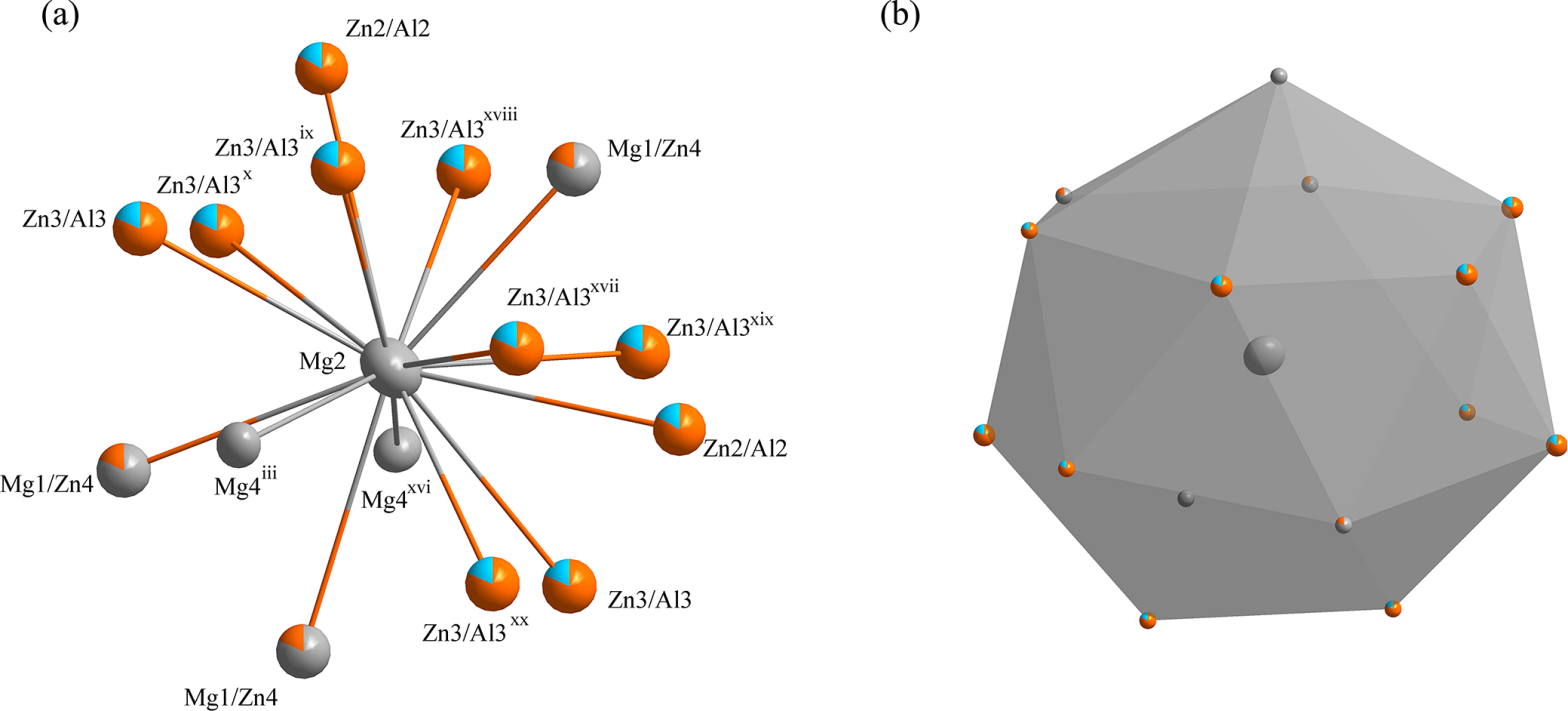
(*a*) The environment of the Mg2 site with displacement ellipsoids given at the 99% probability level; (*b*) the 26-face polyhedron formed around the Mg2 site at the 12*e* site [Symmetry codes: (iii) *z*, *x*, *y*; (ix) −*y* + 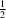, −*z* + 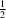, −*x* + 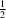; (xvi) *z*, −*x*, −*y* + 1; (xvii) −*y* + 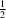, *z* − 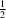, −*x* + 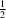; (xviii) −*y* + 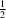, −*z* + 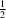, *x* + 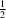; (xix) −*y* + 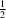, *z* − 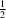, *x* + 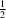; (xx) *x*, −*y*, *z*].

**Figure 3:**
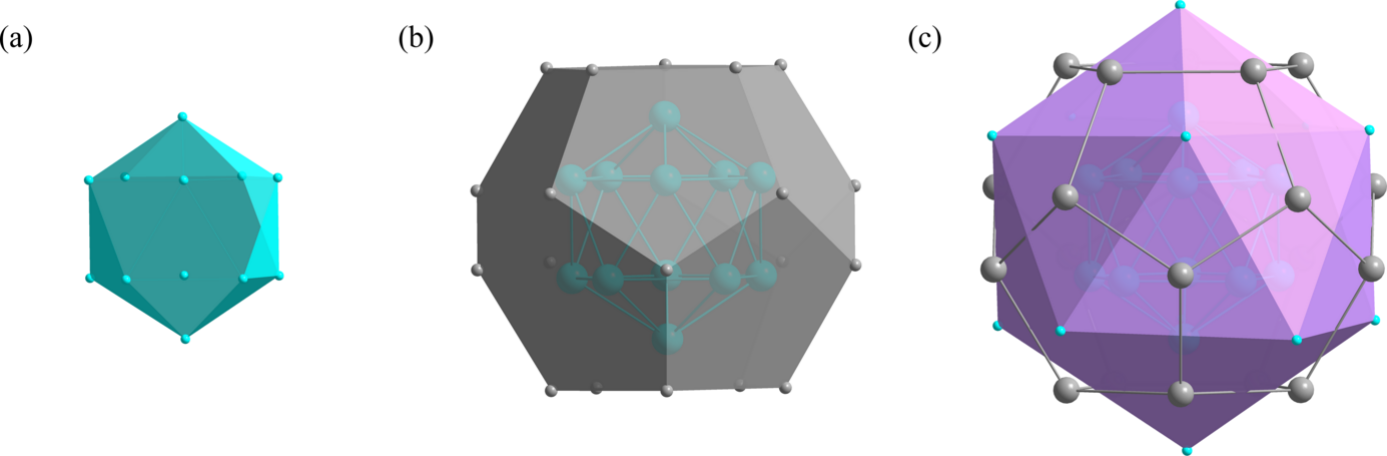
The polyhedra around the 2*a* site with increasing shell size.

**Figure 4:**
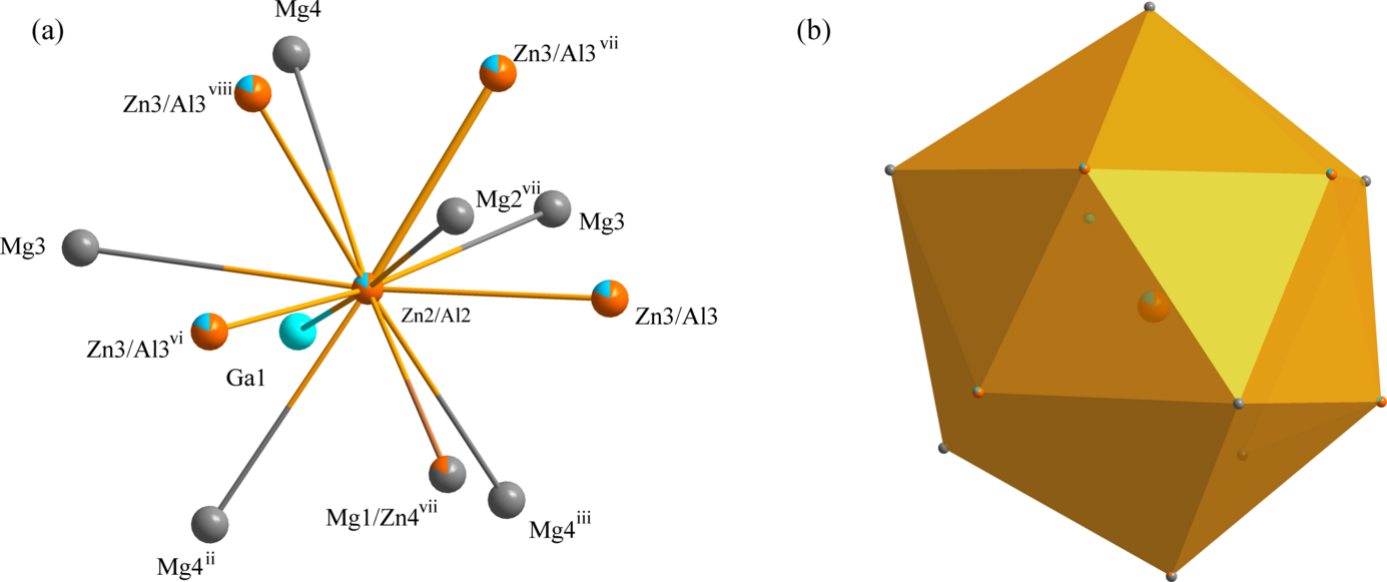
(*a*) The environment of the Zn2/Al2 atom with displacement ellipsoids given at the 99% probability level; (*b*) The icosa­hedron formed around the Zn2/Al2 atom at the 24*g* site [Symmetry codes: (ii) −*z*, *x*, *y*; (iii) *z*, *x*, *y*; (vi) −*x*, *y*, *z*; (vii) −*z* + 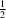, −*x* + 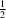, −*y* + 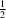; (viii) *z* − 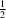, −*x* + 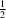, −*y* + 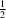].

In the present study, a cubic phase with *a* = 14.1529 (17) Å in space group *Im*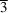 with composition Mg_61.81_Al_12.77_Zn_61.41_Ga_24_ has been discovered and refined on the basis of single-crystal X-ray diffraction, and its chemical composition is in accordance with the EDX results (see the supporting information). The unit cell is illustrated in Fig. 1 ▸. There are seven metal atom sites: one is occupied by gallium, two are co-occupied by aluminium and zinc, another one co-occupied by zinc and magnesium, and three by magnesium. The crystal structure can be described by two kinds of clusters which are a 26-face polyhedron centred at Mg2, and a Bergman cluster centred at a vacancy site. The environments of the Mg2 site is delineated in Fig. 2 ▸. The Mg2 is located at a position with site symmetry *mm*2.. (multiplicity 12, Wyckoff letter *e*) and is surrounded by eight Zn3/Al3 atoms (1, 48*h*), three Mg1/Zn4 atoms (*mm*2.., 12*e*), two Zn2/Al2 atoms (*m*.., 24*g*), and two Mg4 atoms (*m*.., 24*g*). The typical shelled Bergman cluster centred at a virtual non-occupied 2*a* position includes 12 atoms in the first shell, 20 atoms in the second shell, and 12 atoms in the third shell. The environments of the 2*a* sites are delineated in Fig. 3 ▸. The first shell consists of twelve Ga1 atoms (*m*.., 24*g*), the second shell of twelve Mg4 atoms (*m*.., 24*g*) and eight Mg3 atoms (.3., 16*f*), and the third shell of twelve Zn2/Al2 atoms (*m*.., 24*g*). The crystal structure also can be described by one cluster which is an icosa­hedral cluster centred at Zn2/Al2. The environment of the Zn2/Al2 site is delineated in Fig. 4 ▸. The central Zn2/Al2 site is surrounded by one Ga1 atom (*m*.., 24*g*), four Zn3/Al3 atoms (1, 48*h*), one Mg1/Zn4 atom (*mm*2.., 12*e*), one Mg2 atom (*mm*2.., 12*e*), two Mg3 atoms (.3., 16*f*), and three Mg4 atoms (*m*.., 24*g*).

The structure described in this paper shares similarities with two previously reported crystal structures in terms of their basic framework. However, there are also significant differences. To compare with the crystal structure model reported by Bergman *et al.* (1957 ▸): (i) There are no atoms occupying the 2*a* position in the present model, while it is occupied by a vacancy aluminium atom in their model; (ii) in the present model, the 12*e* position is co-occupied by zinc and aluminium atoms while it is solely occupied by one magnesium atom in the previous model; (iii) a gallium atom occupies a 24*g* position in the present model, while there is no atom at the same position in Bergman *et al.*’s model. To compare with another previously reported structure (Montagné & Tillard, 2016 ▸), the 24*g* position is jointly occupied by aluminium and zinc atoms, while it is only occupied by a gallium atom in the present refined model, and one aluminium atom occupies one of the 12*e* positions. However, the structure delineated in this paper deviates from the aforementioned positions in the following ways: firstly, it is devoid of an additional 2*a* position; secondly, the 12*e* position is occupied by zinc and aluminium atoms. Additionally, a gallium atom occupies a 24*g* position. In the previously reported structure (Montagné & Tillard, 2016 ▸) the 24*g* position was jointly occupied by aluminium and zinc atoms. In contrast, in the crystal structure described in this paper, this position is occupied by a gallium atom.

## Synthesis and crystallization

High-purity magnesium (99.90% purity; 0.2186 g), aluminium (99.95% purity; 0.0882 g), gallium (99.90% purity; 0.0976 g) and zinc (99.90% purity; 0.5955 g) were mixed evenly and ground well in an agate mortar. Subsequently, the blended powder was placed in a carbide grinding die with a diameter of 5 mm and pressed into a tablet at approximately 4 MPa for one minute. The resulting material was a cylindrical block that exhibited no signs of deformation or cracking. Further details regarding the high-pressure sinter­ing experiment utilizing the 1-hexa­nol high-temperature and high-pressure apparatus can be found in elsewhere (Liu & Fan, 2018 ▸). The sample was subjected to a pressure of 4 GPa and heated to a temperature of 1073 K for a period of 30 minutes, before being rapidly cooled to room temperature by the deactivation of the furnace power. A single crystal was selected and mounted on a glass fibre for SXRD measurements.

## Refinement

Crystal data, data collection and structure refinement details are summarized in Table 1 ▸. Occupancies for atoms sharing the same site were refined: Zn2 and Al2 atoms have site occupancies of 0.833 (15) and 0.167 (15); Zn3 and Al3 atoms coexist in a position with occupancies 0.817 (12) and 0.183 (12); while Mg1 and Zn4 atoms coexist in a position with occupancies 0.818 (16) and 0.182 (16), respectively.

## Supplementary Material

Crystal structure: contains datablock(s) I. DOI: 10.1107/S2414314625007709/bh4098sup1.cif

Structure factors: contains datablock(s) I. DOI: 10.1107/S2414314625007709/bh4098Isup2.hkl

Supporting information file. DOI: 10.1107/S2414314625007709/bh4098sup3.docx

CCDC reference: 2483585

Additional supporting information:  crystallographic information; 3D view; checkCIF report

Additional supporting information:  crystallographic information; 3D view; checkCIF report

## Figures and Tables

**Table 1:** Experimental details

Crystal data	
Chemical formula	Mg_61.81_Al_12.77_Zn_61.42_Ga_24_
*M*_r_	7535.29
Crystal system, space group	Cubic, *Im* 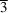
Temperature (K)	296
*a* (Å)	14.1529 (17)
*V* (Å^3^)	2834.9 (10)
*Z*	1
Radiation type	Mo *K*α
μ (mm^−1^)	18.75
Crystal size (mm)	0.18 × 0.12 × 0.06

Data collection	
Diffractometer	Bruker D8 Venture Photon 100 CMOS
Absorption correction	Multi-scan (*SADABS*; Krause *et al.*, 2015 ▸)
*T*_min_, *T*_max_	0.496, 0.523
No. of measured, independent and observed [*I* > 2σ(*I*)] reflections	7679, 475, 341
*R*_int_	0.152
(sin θ/λ)_max_ (Å^−1^)	0.593

Refinement	
*R*[*F*^2^ > 2σ(*F*^2^)], *wR*(*F*^2^), *S*	0.059, 0.105, 1.14
No. of reflections	475
No. of parameters	42
Δρ_max_, Δρ_min_ (e Å^−3^)	1.31, −0.83

Computer programs: *APEX5* and *SAINT* (Bruker, 2023 ▸), *SHELXT2018/2* (Sheldrick, 2015*a* ▸), *SHELXL2018/3* (Sheldrick, 2015*b* ▸), *DIAMOND* (Brandenburg & Putz, 2017 ▸) and *publCIF* (Westrip, 2010 ▸).

## full crystallographic data

### Dohexacontamagnesium tridecaaluminium henihexacontazinc tetracosagallium Crystal data

**Table:**

Mg_61.81_Al_12.77_Zn_61.42_Ga_24_	Mo *K*α radiation, λ = 0.71073 Å
*M**_r_* = 7535.29	Cell parameters from 2565 reflections
Cubic, *I**m*3	θ = 3.2–27.0°
*a* = 14.1529 (17) Å	µ = 18.75 mm^−^^1^
*V* = 2834.9 (10) Å^3^	*T* = 296 K
*Z* = 1	Lump, grey
*F*(000) = 3494	0.18 × 0.12 × 0.06 mm
*D*_x_ = 4.414 Mg m^−^^3^	

### Dohexacontamagnesium tridecaaluminium henihexacontazinc tetracosagallium Data collection

**Table:**

Bruker D8 Venture Photon 100 CMOS diffractometer	341 reflections with *I* > 2σ(*I*)
phi and ω scans	*R*_int_ = 0.152
Absorption correction: multi-scan (SADABS; Krause *et al.*, 2015)	θ_max_ = 24.9°, θ_min_ = 2.9°
*T*_min_ = 0.496, *T*_max_ = 0.523	*h* = −15→16
7679 measured reflections	*k* = −16→11
475 independent reflections	*l* = −16→16

### Dohexacontamagnesium tridecaaluminium henihexacontazinc tetracosagallium Refinement

**Table:**

Refinement on *F*^2^	0 restraints
Least-squares matrix: full	Primary atom site location: dual
*R*[*F*^2^ > 2σ(*F*^2^)] = 0.059	*w* = 1/[σ^2^(*F*_o_^2^) + (0.0311*P*)^2^ + 65.7834*P*] where *P* = (*F*_o_^2^ + 2*F*_c_^2^)/3
*wR*(*F*^2^) = 0.105	(Δ/σ)_max_ < 0.001
*S* = 1.14	Δρ_max_ = 1.31 e Å^−^^3^
475 reflections	Δρ_min_ = −0.83 e Å^−^^3^
42 parameters	

### Dohexacontamagnesium tridecaaluminium henihexacontazinc tetracosagallium Fractional atomic coordinates and isotropic or equivalent isotropic displacement parameters (Å^2^)

**Table:**

	*x*	*y*	*z*	*U*_iso_*/*U*_eq_	Occ. (<1)
Ga1	0.000000	0.09285 (13)	0.15109 (12)	0.0124 (7)	
Zn2	0.000000	0.17895 (15)	0.30668 (15)	0.0127 (8)	0.833 (15)
Al2	0.000000	0.17895 (15)	0.30668 (15)	0.0127 (8)	0.167 (15)
Zn3	0.15792 (11)	0.19038 (10)	0.40335 (10)	0.0134 (6)	0.817 (12)
Al3	0.15792 (11)	0.19038 (10)	0.40335 (10)	0.0134 (6)	0.183 (12)
Mg1	0.4030 (4)	0.000000	0.500000	0.014 (2)	0.818 (16)
Zn4	0.4030 (4)	0.000000	0.500000	0.014 (2)	0.182 (16)
Mg2	0.1989 (6)	0.000000	0.500000	0.019 (2)	
Mg3	0.1861 (3)	0.1861 (3)	0.1861 (3)	0.0126 (17)	
Mg4	0.000000	0.3005 (4)	0.1170 (4)	0.0104 (14)	

### Dohexacontamagnesium tridecaaluminium henihexacontazinc tetracosagallium Atomic displacement parameters (Å^2^)

**Table:**

	*U*^11^	*U*^22^	*U*^33^	*U*^12^	*U*^13^	*U*^23^
Ga1	0.0134 (11)	0.0112 (12)	0.0125 (12)	0.000	0.000	−0.0002 (8)
Zn2	0.0151 (14)	0.0119 (14)	0.0109 (13)	0.000	0.000	−0.0025 (10)
Al2	0.0151 (14)	0.0119 (14)	0.0109 (13)	0.000	0.000	−0.0025 (10)
Zn3	0.0108 (10)	0.0152 (10)	0.0142 (10)	0.0009 (7)	−0.0005 (7)	0.0004 (7)
Al3	0.0108 (10)	0.0152 (10)	0.0142 (10)	0.0009 (7)	−0.0005 (7)	0.0004 (7)
Mg1	0.009 (4)	0.020 (4)	0.012 (4)	0.000	0.000	0.000
Zn4	0.009 (4)	0.020 (4)	0.012 (4)	0.000	0.000	0.000
Mg2	0.013 (5)	0.021 (5)	0.022 (5)	0.000	0.000	0.000
Mg3	0.0126 (17)	0.0126 (17)	0.0126 (17)	−0.0002 (17)	−0.0002 (17)	−0.0002 (17)
Mg4	0.009 (3)	0.011 (3)	0.010 (3)	0.000	0.000	−0.002 (2)

### Dohexacontamagnesium tridecaaluminium henihexacontazinc tetracosagallium Geometric parameters (Å, º)

**Table:**

Ga1—Al2	2.517 (3)	Al2—Mg2^vii^	3.236 (5)
Ga1—Zn2	2.517 (3)	Zn3—Zn3^ix^	2.6686 (15)
Ga1—Ga1^i^	2.628 (4)	Zn3—Zn3^vii^	2.6687 (15)
Ga1—Ga1^ii^	2.642 (2)	Zn3—Zn3^x^	2.736 (3)
Ga1—Ga1^iii^	2.642 (2)	Zn3—Mg1^vii^	2.934 (3)
Ga1—Ga1^iv^	2.642 (2)	Zn3—Mg2^vii^	3.053 (4)
Ga1—Ga1^v^	2.642 (2)	Zn3—Mg2	3.077 (2)
Ga1—Mg4	2.978 (6)	Zn3—Mg3^xi^	3.088 (4)
Ga1—Mg3^vi^	2.987 (6)	Zn3—Mg3	3.101 (4)
Ga1—Mg3	2.987 (6)	Zn3—Mg4^ix^	3.106 (5)
Ga1—Mg4^iii^	2.991 (5)	Zn3—Mg4^iii^	3.117 (3)
Ga1—Mg4^ii^	2.991 (5)	Al3—Mg2^vii^	3.053 (4)
Zn2—Zn3^vi^	2.6255 (19)	Al3—Mg2	3.077 (2)
Zn2—Zn3	2.6255 (19)	Al3—Mg3^xi^	3.088 (4)
Zn2—Zn3^vii^	2.684 (2)	Al3—Mg3	3.101 (4)
Zn2—Zn3^viii^	2.684 (2)	Al3—Mg4^ix^	3.106 (5)
Zn2—Mg1^vii^	2.971 (3)	Al3—Mg4^iii^	3.117 (3)
Zn2—Mg4^ii^	3.028 (3)	Mg1—Mg1^xii^	2.747 (11)
Zn2—Mg4^iii^	3.028 (3)	Mg1—Mg2	2.888 (10)
Zn2—Mg3^vi^	3.1400 (17)	Mg1—Mg2^xiii^	3.132 (8)
Zn2—Mg3	3.1400 (17)	Mg1—Mg2^ix^	3.132 (8)
Zn2—Mg4	3.188 (6)	Mg1—Mg4^xiv^	3.550 (5)
Zn2—Mg2^vii^	3.236 (5)	Mg1—Mg4^xv^	3.550 (5)
Al2—Al3	2.6255 (19)	Zn4—Mg2	2.888 (10)
Al2—Mg4^ii^	3.028 (3)	Zn4—Mg2^xiii^	3.132 (8)
Al2—Mg4^iii^	3.028 (3)	Zn4—Mg2^ix^	3.132 (8)
Al2—Mg3^vi^	3.1400 (17)	Mg2—Mg4^iii^	3.052 (6)
Al2—Mg3	3.1400 (17)	Mg2—Mg4^xvi^	3.052 (6)
Al2—Mg4	3.188 (6)	Mg3—Mg3^xi^	3.133 (13)
			
Al2—Ga1—Ga1^i^	118.96 (6)	Mg1^xii^—Mg1—Mg2	180.0
Zn2—Ga1—Ga1^i^	118.96 (6)	Mg1^xii^—Mg1—Zn3^xvii^	116.75 (10)
Al2—Ga1—Ga1^ii^	120.92 (4)	Mg2—Mg1—Zn3^xvii^	63.25 (10)
Zn2—Ga1—Ga1^ii^	120.92 (4)	Mg1^xii^—Mg1—Zn3^xviii^	116.75 (10)
Ga1^i^—Ga1—Ga1^ii^	60.17 (5)	Mg2—Mg1—Zn3^xviii^	63.25 (10)
Al2—Ga1—Ga1^iii^	120.92 (4)	Zn3^xvii^—Mg1—Zn3^xviii^	126.5 (2)
Zn2—Ga1—Ga1^iii^	120.92 (4)	Mg1^xii^—Mg1—Zn3^ix^	116.75 (10)
Ga1^i^—Ga1—Ga1^iii^	60.17 (5)	Mg2—Mg1—Zn3^ix^	63.25 (10)
Ga1^ii^—Ga1—Ga1^iii^	108.08 (2)	Zn3^xvii^—Mg1—Zn3^ix^	55.56 (8)
Al2—Ga1—Ga1^iv^	123.86 (6)	Zn3^xviii^—Mg1—Zn3^ix^	99.22 (13)
Zn2—Ga1—Ga1^iv^	123.86 (6)	Mg1^xii^—Mg1—Zn3^xix^	116.75 (10)
Ga1^i^—Ga1—Ga1^iv^	108.18 (5)	Mg2—Mg1—Zn3^xix^	63.25 (10)
Ga1^ii^—Ga1—Ga1^iv^	60.0	Zn3^xvii^—Mg1—Zn3^xix^	99.22 (13)
Ga1^iii^—Ga1—Ga1^iv^	107.78 (6)	Zn3^xviii^—Mg1—Zn3^xix^	55.56 (8)
Al2—Ga1—Ga1^v^	123.86 (6)	Zn3^ix^—Mg1—Zn3^xix^	126.5 (2)
Zn2—Ga1—Ga1^v^	123.86 (6)	Mg1^xii^—Mg1—Zn2^xvii^	112.96 (11)
Ga1^i^—Ga1—Ga1^v^	108.18 (5)	Mg2—Mg1—Zn2^xvii^	67.04 (11)
Ga1^ii^—Ga1—Ga1^v^	107.78 (6)	Zn3^xvii^—Mg1—Zn2^xvii^	52.79 (6)
Ga1^iii^—Ga1—Ga1^v^	60.0	Zn3^xviii^—Mg1—Zn2^xvii^	104.69 (12)
Ga1^iv^—Ga1—Ga1^v^	59.66 (9)	Zn3^ix^—Mg1—Zn2^xvii^	104.69 (12)
Al2—Ga1—Mg4	70.35 (12)	Zn3^xix^—Mg1—Zn2^xvii^	52.79 (6)
Zn2—Ga1—Mg4	70.35 (12)	Mg1^xii^—Mg1—Zn2^ix^	112.96 (11)
Ga1^i^—Ga1—Mg4	170.69 (11)	Mg2—Mg1—Zn2^ix^	67.04 (11)
Ga1^ii^—Ga1—Mg4	116.13 (7)	Zn3^xvii^—Mg1—Zn2^ix^	104.69 (12)
Ga1^iii^—Ga1—Mg4	116.13 (7)	Zn3^xviii^—Mg1—Zn2^ix^	52.79 (6)
Ga1^iv^—Ga1—Mg4	63.97 (11)	Zn3^ix^—Mg1—Zn2^ix^	52.79 (6)
Ga1^v^—Ga1—Mg4	63.97 (11)	Zn3^xix^—Mg1—Zn2^ix^	104.69 (12)
Zn2—Ga1—Mg3^vi^	68.96 (7)	Zn2^xvii^—Mg1—Zn2^ix^	134.1 (2)
Ga1^i^—Ga1—Mg3^vi^	116.22 (4)	Mg1^xii^—Mg1—Mg2^xiii^	63.99 (11)
Ga1^ii^—Ga1—Mg3^vi^	63.76 (6)	Mg2—Mg1—Mg2^xiii^	116.01 (11)
Ga1^iii^—Ga1—Mg3^vi^	170.12 (8)	Zn3^xvii^—Mg1—Mg2^xiii^	151.87 (3)
Ga1^iv^—Ga1—Mg3^vi^	63.76 (6)	Zn3^xviii^—Mg1—Mg2^xiii^	60.85 (6)
Ga1^v^—Ga1—Mg3^vi^	115.78 (10)	Zn3^ix^—Mg1—Mg2^xiii^	151.87 (3)
Mg4—Ga1—Mg3^vi^	65.85 (4)	Zn3^xix^—Mg1—Mg2^xiii^	60.85 (6)
Al2—Ga1—Mg3	68.96 (7)	Zn2^xvii^—Mg1—Mg2^xiii^	99.85 (3)
Zn2—Ga1—Mg3	68.96 (7)	Zn2^ix^—Mg1—Mg2^xiii^	99.85 (3)
Ga1^i^—Ga1—Mg3	116.22 (4)	Mg1^xii^—Mg1—Mg2^ix^	63.99 (11)
Ga1^ii^—Ga1—Mg3	170.12 (8)	Mg2—Mg1—Mg2^ix^	116.01 (11)
Ga1^iii^—Ga1—Mg3	63.76 (6)	Zn3^xvii^—Mg1—Mg2^ix^	60.85 (6)
Ga1^iv^—Ga1—Mg3	115.79 (10)	Zn3^xviii^—Mg1—Mg2^ix^	151.87 (3)
Ga1^v^—Ga1—Mg3	63.76 (6)	Zn3^ix^—Mg1—Mg2^ix^	60.85 (6)
Mg4—Ga1—Mg3	65.85 (4)	Zn3^xix^—Mg1—Mg2^ix^	151.87 (3)
Mg3^vi^—Ga1—Mg3	123.69 (11)	Zn2^xvii^—Mg1—Mg2^ix^	99.85 (3)
Al2—Ga1—Mg4^iii^	66.05 (9)	Zn2^ix^—Mg1—Mg2^ix^	99.85 (3)
Zn2—Ga1—Mg4^iii^	66.05 (9)	Mg2^xiii^—Mg1—Mg2^ix^	128.0 (2)
Ga1^i^—Ga1—Mg4^iii^	63.93 (5)	Mg1^xii^—Mg1—Mg4^xiv^	67.24 (9)
Ga1^ii^—Ga1—Mg4^iii^	116.77 (11)	Mg2—Mg1—Mg4^xiv^	112.76 (9)
Ga1^iii^—Ga1—Mg4^iii^	63.49 (11)	Zn3^xvii^—Mg1—Mg4^xiv^	154.20 (9)
Ga1^iv^—Ga1—Mg4^iii^	170.08 (8)	Zn3^xviii^—Mg1—Mg4^xiv^	56.50 (6)
Ga1^v^—Ga1—Mg4^iii^	115.72 (11)	Zn3^ix^—Mg1—Mg4^xiv^	99.14 (8)
Mg4—Ga1—Mg4^iii^	123.23 (13)	Zn3^xix^—Mg1—Mg4^xiv^	100.92 (8)
Mg3^vi^—Ga1—Mg4^iii^	124.42 (13)	Zn2^xvii^—Mg1—Mg4^xiv^	152.04 (8)
Mg3—Ga1—Mg4^iii^	65.70 (8)	Zn2^ix^—Mg1—Mg4^xiv^	54.45 (7)
Al2—Ga1—Mg4^ii^	66.05 (9)	Mg2^xiii^—Mg1—Mg4^xiv^	53.91 (9)
Zn2—Ga1—Mg4^ii^	66.05 (9)	Mg2^ix^—Mg1—Mg4^xiv^	104.46 (13)
Ga1^i^—Ga1—Mg4^ii^	63.93 (5)	Mg1^xii^—Mg1—Mg4^xv^	67.24 (9)
Ga1^ii^—Ga1—Mg4^ii^	63.49 (11)	Mg2—Mg1—Mg4^xv^	112.76 (9)
Ga1^iii^—Ga1—Mg4^ii^	116.77 (11)	Zn3^xvii^—Mg1—Mg4^xv^	56.50 (6)
Ga1^iv^—Ga1—Mg4^ii^	115.72 (11)	Zn3^xviii^—Mg1—Mg4^xv^	154.20 (9)
Ga1^v^—Ga1—Mg4^ii^	170.08 (8)	Zn3^ix^—Mg1—Mg4^xv^	100.92 (8)
Mg4—Ga1—Mg4^ii^	123.23 (13)	Zn3^xix^—Mg1—Mg4^xv^	99.14 (8)
Mg3^vi^—Ga1—Mg4^ii^	65.70 (8)	Zn2^xvii^—Mg1—Mg4^xv^	54.45 (7)
Mg3—Ga1—Mg4^ii^	124.42 (13)	Zn2^ix^—Mg1—Mg4^xv^	152.04 (8)
Mg4^iii^—Ga1—Mg4^ii^	67.3 (2)	Mg2^xiii^—Mg1—Mg4^xv^	104.46 (13)
Ga1—Zn2—Zn3^vi^	119.07 (6)	Mg2^ix^—Mg1—Mg4^xv^	53.91 (9)
Ga1—Zn2—Zn3	119.07 (6)	Mg4^xiv^—Mg1—Mg4^xv^	134.48 (18)
Zn3^vi^—Zn2—Zn3	116.69 (11)	Mg2—Zn4—Mg2^xiii^	116.01 (11)
Ga1—Zn2—Zn3^vii^	115.47 (8)	Mg2—Zn4—Mg2^ix^	116.01 (11)
Zn3^vi^—Zn2—Zn3^vii^	111.88 (9)	Mg2^xiii^—Zn4—Mg2^ix^	128.0 (2)
Zn3—Zn2—Zn3^vii^	60.34 (5)	Zn4—Mg2—Mg4^iii^	112.31 (17)
Ga1—Zn2—Zn3^viii^	115.47 (8)	Mg1—Mg2—Mg4^iii^	112.31 (17)
Zn3^vi^—Zn2—Zn3^viii^	60.34 (5)	Zn4—Mg2—Mg4^xvi^	112.31 (17)
Zn3—Zn2—Zn3^viii^	111.88 (9)	Mg1—Mg2—Mg4^xvi^	112.31 (17)
Zn3^vii^—Zn2—Zn3^viii^	61.28 (8)	Mg4^iii^—Mg2—Mg4^xvi^	135.4 (3)
Ga1—Zn2—Mg1^vii^	128.08 (13)	Mg1—Mg2—Zn3^xviii^	59.12 (13)
Zn3^vi^—Zn2—Mg1^vii^	62.88 (6)	Mg4^iii^—Mg2—Zn3^xviii^	150.70 (9)
Zn3—Zn2—Mg1^vii^	62.88 (6)	Mg4^xvi^—Mg2—Zn3^xviii^	61.16 (9)
Zn3^vii^—Zn2—Mg1^vii^	108.74 (11)	Zn4—Mg2—Zn3^xix^	59.12 (13)
Zn3^viii^—Zn2—Mg1^vii^	108.74 (11)	Mg1—Mg2—Zn3^xix^	59.12 (13)
Ga1—Zn2—Mg4^ii^	64.52 (11)	Mg4^iii^—Mg2—Zn3^xix^	150.70 (9)
Zn3^vi^—Zn2—Mg4^ii^	66.47 (10)	Mg4^xvi^—Mg2—Zn3^xix^	61.16 (9)
Zn3—Zn2—Mg4^ii^	122.16 (12)	Zn3^xviii^—Mg2—Zn3^xix^	53.23 (9)
Zn3^vii^—Zn2—Mg4^ii^	177.34 (11)	Zn4—Mg2—Zn3^xvii^	59.12 (13)
Zn3^viii^—Zn2—Mg4^ii^	116.18 (9)	Mg1—Mg2—Zn3^xvii^	59.12 (13)
Mg1^vii^—Zn2—Mg4^ii^	72.55 (13)	Mg4^iii^—Mg2—Zn3^xvii^	61.16 (9)
Ga1—Zn2—Mg4^iii^	64.52 (11)	Mg4^xvi^—Mg2—Zn3^xvii^	150.70 (9)
Zn3^vi^—Zn2—Mg4^iii^	122.16 (12)	Zn3^xviii^—Mg2—Zn3^xvii^	118.2 (3)
Zn3—Zn2—Mg4^iii^	66.47 (10)	Zn3^xix^—Mg2—Zn3^xvii^	94.12 (17)
Zn3^vii^—Zn2—Mg4^iii^	116.18 (9)	Zn4—Mg2—Zn3^ix^	59.12 (13)
Zn3^viii^—Zn2—Mg4^iii^	177.34 (11)	Mg1—Mg2—Zn3^ix^	59.12 (13)
Mg1^vii^—Zn2—Mg4^iii^	72.55 (13)	Mg4^iii^—Mg2—Zn3^ix^	61.16 (9)
Mg4^ii^—Zn2—Mg4^iii^	66.35 (18)	Mg4^xvi^—Mg2—Zn3^ix^	150.70 (9)
Ga1—Zn2—Mg3^vi^	62.62 (12)	Zn3^xviii^—Mg2—Zn3^ix^	94.12 (17)
Zn3^vi^—Zn2—Mg3^vi^	64.36 (10)	Zn3^xix^—Mg2—Zn3^ix^	118.2 (3)
Zn3—Zn2—Mg3^vi^	174.43 (10)	Zn3^xvii^—Mg2—Zn3^ix^	53.23 (9)
Zn3^vii^—Zn2—Mg3^vi^	114.09 (8)	Mg1—Mg2—Zn3	100.87 (15)
Zn3^viii^—Zn2—Mg3^vi^	63.46 (10)	Mg4^iii^—Mg2—Zn3	61.13 (6)
Mg1^vii^—Zn2—Mg3^vi^	120.86 (7)	Mg4^xvi^—Mg2—Zn3	109.86 (13)
Mg4^ii^—Zn2—Mg3^vi^	63.41 (10)	Zn3^xviii^—Mg2—Zn3	91.71 (5)
Mg4^iii^—Zn2—Mg3^vi^	118.05 (16)	Zn3^xix^—Mg2—Zn3	144.54 (16)
Ga1—Zn2—Mg3	62.62 (12)	Zn3^xvii^—Mg2—Zn3	99.42 (6)
Zn3^vi^—Zn2—Mg3	174.42 (10)	Zn3^ix^—Mg2—Zn3	51.61 (5)
Zn3—Zn2—Mg3	64.36 (10)	Zn4—Mg2—Al3	100.87 (15)
Zn3^vii^—Zn2—Mg3	63.46 (10)	Mg4^iii^—Mg2—Al3	61.13 (6)
Zn3^viii^—Zn2—Mg3	114.09 (8)	Mg4^xvi^—Mg2—Al3	109.86 (13)
Mg1^vii^—Zn2—Mg3	120.86 (7)	Mg1—Mg2—Zn3^xx^	100.87 (15)
Mg4^ii^—Zn2—Mg3	118.05 (15)	Mg4^iii^—Mg2—Zn3^xx^	61.13 (6)
Mg4^iii^—Zn2—Mg3	63.41 (10)	Mg4^xvi^—Mg2—Zn3^xx^	109.86 (13)
Mg3^vi^—Zn2—Mg3	114.0 (2)	Zn3^xviii^—Mg2—Zn3^xx^	144.54 (16)
Ga1—Zn2—Mg4	61.62 (11)	Zn3^xix^—Mg2—Zn3^xx^	91.71 (5)
Zn3^vi^—Zn2—Mg4	113.92 (7)	Zn3^xvii^—Mg2—Zn3^xx^	51.61 (5)
Zn3—Zn2—Mg4	113.92 (7)	Zn3^ix^—Mg2—Zn3^xx^	99.42 (6)
Zn3^vii^—Zn2—Mg4	63.18 (9)	Zn3—Mg2—Zn3^xx^	122.25 (11)
Zn3^viii^—Zn2—Mg4	63.18 (9)	Mg1—Mg2—Zn3^x^	100.87 (15)
Mg1^vii^—Zn2—Mg4	170.30 (16)	Mg4^iii^—Mg2—Zn3^x^	109.86 (13)
Mg4^ii^—Zn2—Mg4	115.29 (17)	Mg4^xvi^—Mg2—Zn3^x^	61.13 (6)
Mg4^iii^—Zn2—Mg4	115.29 (17)	Zn3^xviii^—Mg2—Zn3^x^	51.61 (5)
Mg3^vi^—Zn2—Mg4	61.64 (6)	Zn3^xix^—Mg2—Zn3^x^	99.42 (6)
Mg3—Zn2—Mg4	61.65 (6)	Zn3^xvii^—Mg2—Zn3^x^	144.54 (16)
Ga1—Zn2—Mg2^vii^	176.67 (15)	Zn3^ix^—Mg2—Zn3^x^	91.71 (5)
Zn3^vi^—Zn2—Mg2^vii^	61.74 (6)	Zn3—Mg2—Zn3^x^	52.79 (6)
Zn3—Zn2—Mg2^vii^	61.74 (6)	Zn3^xx^—Mg2—Zn3^x^	158.3 (3)
Zn3^vii^—Zn2—Mg2^vii^	61.80 (11)	Ga1^v^—Mg3—Ga1^iii^	52.48 (12)
Zn3^viii^—Zn2—Mg2^vii^	61.80 (11)	Ga1^v^—Mg3—Ga1	52.48 (12)
Mg1^vii^—Zn2—Mg2^vii^	55.25 (16)	Ga1^iii^—Mg3—Ga1	52.48 (12)
Mg4^ii^—Zn2—Mg2^vii^	118.11 (14)	Ga1^v^—Mg3—Zn3^ix^	144.34 (14)
Mg4^iii^—Zn2—Mg2^vii^	118.11 (14)	Ga1^iii^—Mg3—Zn3^ix^	92.77 (6)
Mg3^vi^—Zn2—Mg2^vii^	116.25 (13)	Ga1—Mg3—Zn3^ix^	115.89 (8)
Mg3—Zn2—Mg2^vii^	116.25 (13)	Ga1^v^—Mg3—Zn3^xi^	92.77 (6)
Mg4—Zn2—Mg2^vii^	115.05 (17)	Ga1^iii^—Mg3—Zn3^xi^	115.89 (8)
Ga1—Al2—Al3	119.07 (6)	Ga1—Mg3—Zn3^xi^	144.34 (14)
Ga1—Al2—Mg4^ii^	64.52 (11)	Zn3^ix^—Mg3—Zn3^xi^	96.92 (14)
Al3—Al2—Mg4^ii^	122.16 (12)	Ga1^v^—Mg3—Zn3^vii^	115.89 (8)
Ga1—Al2—Mg4^iii^	64.52 (11)	Ga1^iii^—Mg3—Zn3^vii^	144.34 (14)
Al3—Al2—Mg4^iii^	66.47 (10)	Ga1—Mg3—Zn3^vii^	92.77 (6)
Mg4^ii^—Al2—Mg4^iii^	66.35 (18)	Zn3^ix^—Mg3—Zn3^vii^	96.92 (14)
Ga1—Al2—Mg3^vi^	62.62 (12)	Zn3^xi^—Mg3—Zn3^vii^	96.92 (14)
Mg4^ii^—Al2—Mg3^vi^	63.41 (10)	Ga1^v^—Mg3—Zn3	145.14 (14)
Mg4^iii^—Al2—Mg3^vi^	118.05 (16)	Ga1^iii^—Mg3—Zn3	115.72 (8)
Ga1—Al2—Mg3	62.62 (12)	Ga1—Mg3—Zn3	93.42 (6)
Al3—Al2—Mg3	64.36 (10)	Zn3^ix^—Mg3—Zn3	51.09 (6)
Mg4^ii^—Al2—Mg3	118.05 (15)	Zn3^xi^—Mg3—Zn3	119.2 (2)
Mg4^iii^—Al2—Mg3	63.41 (10)	Zn3^vii^—Mg3—Zn3	51.09 (6)
Mg3^vi^—Al2—Mg3	114.0 (2)	Ga1^v^—Mg3—Al3	145.14 (14)
Ga1—Al2—Mg4	61.62 (11)	Ga1^iii^—Mg3—Al3	115.72 (8)
Al3—Al2—Mg4	113.92 (7)	Ga1—Mg3—Al3	93.42 (6)
Mg4^ii^—Al2—Mg4	115.29 (17)	Ga1^v^—Mg3—Zn3^iii^	115.72 (8)
Mg4^iii^—Al2—Mg4	115.29 (17)	Ga1^iii^—Mg3—Zn3^iii^	93.42 (6)
Mg3^vi^—Al2—Mg4	61.64 (6)	Ga1—Mg3—Zn3^iii^	145.14 (14)
Mg3—Al2—Mg4	61.65 (6)	Zn3^ix^—Mg3—Zn3^iii^	51.09 (6)
Ga1—Al2—Mg2^vii^	176.67 (15)	Zn3^xi^—Mg3—Zn3^iii^	51.09 (6)
Al3—Al2—Mg2^vii^	61.74 (6)	Zn3^vii^—Mg3—Zn3^iii^	119.2 (2)
Mg4^ii^—Al2—Mg2^vii^	118.11 (14)	Zn3—Mg3—Zn3^iii^	96.35 (14)
Mg4^iii^—Al2—Mg2^vii^	118.11 (14)	Al3—Mg3—Zn3^iii^	96.35 (14)
Mg3^vi^—Al2—Mg2^vii^	116.25 (13)	Ga1^v^—Mg3—Zn3^v^	93.42 (6)
Mg3—Al2—Mg2^vii^	116.25 (13)	Ga1^iii^—Mg3—Zn3^v^	145.14 (14)
Mg4—Al2—Mg2^vii^	115.05 (17)	Ga1—Mg3—Zn3^v^	115.72 (8)
Zn2—Zn3—Zn3^ix^	119.01 (9)	Zn3^ix^—Mg3—Zn3^v^	119.2 (2)
Zn2—Zn3—Zn3^vii^	60.91 (7)	Zn3^xi^—Mg3—Zn3^v^	51.09 (6)
Zn3^ix^—Zn3—Zn3^vii^	119.992 (2)	Zn3^vii^—Mg3—Zn3^v^	51.09 (6)
Zn2—Zn3—Zn2^ix^	177.23 (9)	Zn3—Mg3—Zn3^v^	96.35 (14)
Zn3^ix^—Zn3—Zn2^ix^	58.75 (7)	Al3—Mg3—Zn3^v^	96.35 (14)
Zn3^vii^—Zn3—Zn2^ix^	121.36 (9)	Zn3^iii^—Mg3—Zn3^v^	96.35 (14)
Zn2—Zn3—Zn3^x^	121.41 (5)	Ga1^v^—Mg3—Mg3^xi^	149.30 (7)
Zn3^ix^—Zn3—Zn3^x^	108.96 (5)	Ga1^iii^—Mg3—Mg3^xi^	149.30 (7)
Zn3^vii^—Zn3—Zn3^x^	119.81 (5)	Ga1—Mg3—Mg3^xi^	149.30 (7)
Zn2^ix^—Zn3—Zn3^x^	59.36 (4)	Zn3^ix^—Mg3—Mg3^xi^	59.80 (11)
Zn2—Zn3—Mg1^vii^	64.33 (6)	Zn3^xi^—Mg3—Mg3^xi^	59.80 (11)
Zn3^ix^—Zn3—Mg1^vii^	122.71 (12)	Zn3^vii^—Mg3—Mg3^xi^	59.80 (11)
Zn3^vii^—Zn3—Mg1^vii^	110.27 (10)	Zn3—Mg3—Mg3^xi^	59.37 (11)
Zn2^ix^—Zn3—Mg1^vii^	115.13 (6)	Zn3^iii^—Mg3—Mg3^xi^	59.37 (11)
Zn3^x^—Zn3—Mg1^vii^	62.22 (4)	Zn3^v^—Mg3—Mg3^xi^	59.37 (11)
Zn2—Zn3—Mg2^vii^	69.02 (7)	Ga1^v^—Mg3—Zn2^v^	48.42 (7)
Zn3^ix^—Zn3—Mg2^vii^	171.76 (8)	Ga1^iii^—Mg3—Zn2^v^	95.93 (15)
Zn3^vii^—Zn3—Mg2^vii^	64.65 (11)	Ga1—Mg3—Zn2^v^	94.16 (15)
Zn2^ix^—Zn3—Mg2^vii^	113.17 (7)	Zn3^ix^—Mg3—Zn2^v^	147.23 (14)
Zn3^x^—Zn3—Mg2^vii^	63.38 (5)	Zn3^xi^—Mg3—Zn2^v^	51.05 (5)
Mg1^vii^—Zn3—Mg2^vii^	57.63 (16)	Zn3^vii^—Mg3—Zn2^v^	94.19 (6)
Zn2—Zn3—Mg2	109.77 (14)	Zn3—Mg3—Zn2^v^	144.78 (13)
Zn3^ix^—Zn3—Mg2	63.73 (13)	Al3—Mg3—Zn2^v^	144.78 (13)
Zn3^vii^—Zn3—Mg2	170.67 (15)	Zn3^iii^—Mg3—Zn2^v^	96.79 (6)
Zn2^ix^—Zn3—Mg2	67.97 (13)	Zn3^v^—Mg3—Zn2^v^	49.75 (5)
Zn3^x^—Zn3—Mg2	63.61 (3)	Mg3^xi^—Mg3—Zn2^v^	100.90 (12)
Mg1^vii^—Zn3—Mg2	62.75 (17)	Ga1—Mg4—Ga1^iv^	52.54 (10)
Mg2^vii^—Zn3—Mg2	112.85 (5)	Ga1—Mg4—Ga1^v^	52.54 (10)
Zn2—Zn3—Mg3^xi^	115.46 (11)	Ga1^iv^—Mg4—Ga1^v^	52.13 (11)
Zn3^ix^—Zn3—Mg3^xi^	64.72 (4)	Ga1—Mg4—Zn2^iv^	96.72 (12)
Zn3^vii^—Zn3—Mg3^xi^	64.72 (4)	Ga1^iv^—Mg4—Zn2^iv^	49.44 (8)
Zn2^ix^—Zn3—Mg3^xi^	65.49 (9)	Ga1^v^—Mg4—Zn2^iv^	94.87 (15)
Zn3^x^—Zn3—Mg3^xi^	114.21 (11)	Ga1—Mg4—Zn2^v^	96.72 (12)
Mg1^vii^—Zn3—Mg3^xi^	172.14 (13)	Ga1^iv^—Mg4—Zn2^v^	94.87 (15)
Mg2^vii^—Zn3—Mg3^xi^	114.62 (14)	Ga1^v^—Mg4—Zn2^v^	49.44 (8)
Mg2—Zn3—Mg3^xi^	122.91 (15)	Zn2^iv^—Mg4—Zn2^v^	113.55 (18)
Zn2—Zn3—Mg3	65.89 (9)	Ga1—Mg4—Mg2^v^	148.4 (2)
Zn3^ix^—Zn3—Mg3	64.19 (4)	Ga1^iv^—Mg4—Mg2^v^	149.83 (13)
Zn3^vii^—Zn3—Mg3	64.19 (4)	Ga1^v^—Mg4—Mg2^v^	149.83 (13)
Zn2^ix^—Zn3—Mg3	113.26 (10)	Zn2^iv^—Mg4—Mg2^v^	100.43 (14)
Zn3^x^—Zn3—Mg3	172.53 (7)	Zn2^v^—Mg4—Mg2^v^	100.43 (14)
Mg1^vii^—Zn3—Mg3	123.46 (12)	Ga1—Mg4—Zn3^vii^	92.58 (14)
Mg2^vii^—Zn3—Mg3	123.26 (7)	Ga1^iv^—Mg4—Zn3^vii^	144.45 (19)
Mg2—Zn3—Mg3	113.54 (7)	Ga1^v^—Mg4—Zn3^vii^	115.25 (10)
Mg3^xi^—Zn3—Mg3	60.8 (2)	Zn2^iv^—Mg4—Zn3^vii^	147.47 (14)
Zn2—Zn3—Mg4^ix^	116.41 (11)	Zn2^v^—Mg4—Zn3^vii^	96.10 (6)
Zn3^ix^—Zn3—Mg4^ix^	115.28 (12)	Mg2^v^—Mg4—Zn3^vii^	59.44 (16)
Zn3^vii^—Zn3—Mg4^ix^	64.82 (9)	Ga1—Mg4—Zn3^viii^	92.58 (14)
Zn2^ix^—Zn3—Mg4^ix^	66.36 (11)	Ga1^iv^—Mg4—Zn3^viii^	115.25 (10)
Zn3^x^—Zn3—Mg4^ix^	63.87 (5)	Ga1^v^—Mg4—Zn3^viii^	144.45 (19)
Mg1^vii^—Zn3—Mg4^ix^	109.51 (12)	Zn2^iv^—Mg4—Zn3^viii^	96.10 (6)
Mg2^vii^—Zn3—Mg4^ix^	59.40 (14)	Zn2^v^—Mg4—Zn3^viii^	147.47 (14)
Mg2—Zn3—Mg4^ix^	122.48 (9)	Mg2^v^—Mg4—Zn3^viii^	59.44 (16)
Mg3^xi^—Zn3—Mg4^ix^	63.14 (12)	Zn3^vii^—Mg4—Zn3^viii^	52.26 (10)
Mg3—Zn3—Mg4^ix^	115.43 (11)	Ga1—Mg4—Zn3^v^	115.52 (10)
Zn2—Zn3—Mg4^iii^	62.96 (10)	Ga1^iv^—Mg4—Zn3^v^	144.38 (14)
Zn3^ix^—Zn3—Mg4^iii^	64.39 (12)	Ga1^v^—Mg4—Zn3^v^	93.05 (6)
Zn3^vii^—Zn3—Mg4^iii^	113.77 (11)	Zn2^iv^—Mg4—Zn3^v^	144.22 (19)
Zn2^ix^—Zn3—Mg4^iii^	114.27 (11)	Zn2^v^—Mg4—Zn3^v^	50.57 (5)
Zn3^x^—Zn3—Mg4^iii^	117.84 (9)	Mg2^v^—Mg4—Zn3^v^	59.83 (9)
Mg1^vii^—Zn3—Mg4^iii^	71.77 (13)	Zn3^vii^—Mg4—Zn3^v^	50.79 (7)
Mg2^vii^—Zn3—Mg4^iii^	121.14 (15)	Zn3^viii^—Mg4—Zn3^v^	97.44 (14)
Mg2—Zn3—Mg4^iii^	59.04 (10)	Ga1—Mg4—Zn3^xxi^	115.52 (10)
Mg3^xi^—Zn3—Mg4^iii^	115.51 (14)	Ga1^iv^—Mg4—Zn3^xxi^	93.05 (6)
Mg3—Zn3—Mg4^iii^	62.87 (13)	Ga1^v^—Mg4—Zn3^xxi^	144.38 (14)
Mg4^ix^—Zn3—Mg4^iii^	178.29 (12)	Zn2^iv^—Mg4—Zn3^xxi^	50.57 (5)
Al2—Al3—Mg2^vii^	69.02 (7)	Zn2^v^—Mg4—Zn3^xxi^	144.22 (19)
Al2—Al3—Mg2	109.77 (14)	Mg2^v^—Mg4—Zn3^xxi^	59.83 (9)
Mg2^vii^—Al3—Mg2	112.85 (5)	Zn3^vii^—Mg4—Zn3^xxi^	97.44 (14)
Mg2^vii^—Al3—Mg3^xi^	114.62 (14)	Zn3^viii^—Mg4—Zn3^xxi^	50.79 (7)
Mg2—Al3—Mg3^xi^	122.91 (15)	Zn3^v^—Mg4—Zn3^xxi^	119.66 (18)
Al2—Al3—Mg3	65.89 (9)	Ga1—Mg4—Zn2	48.03 (9)
Mg2^vii^—Al3—Mg3	123.26 (7)	Ga1^iv^—Mg4—Zn2	94.86 (14)
Mg2—Al3—Mg3	113.54 (7)	Ga1^v^—Mg4—Zn2	94.86 (14)
Mg3^xi^—Al3—Mg3	60.8 (2)	Zn2^iv^—Mg4—Zn2	118.44 (10)
Al2—Al3—Mg4^ix^	116.41 (11)	Zn2^v^—Mg4—Zn2	118.44 (10)
Mg2^vii^—Al3—Mg4^ix^	59.40 (14)	Mg2^v^—Mg4—Zn2	100.4 (2)
Mg2—Al3—Mg4^ix^	122.48 (9)	Zn3^vii^—Mg4—Zn2	50.46 (9)
Mg3^xi^—Al3—Mg4^ix^	63.14 (12)	Zn3^viii^—Mg4—Zn2	50.46 (9)
Mg3—Al3—Mg4^ix^	115.43 (11)	Zn3^v^—Mg4—Zn2	95.50 (12)
Al2—Al3—Mg4^iii^	62.96 (10)	Zn3^xxi^—Mg4—Zn2	95.50 (12)
Mg2^vii^—Al3—Mg4^iii^	121.14 (15)	Ga1—Mg4—Al2	48.03 (9)
Mg2—Al3—Mg4^iii^	59.04 (10)	Ga1^iv^—Mg4—Al2	94.86 (14)
Mg3^xi^—Al3—Mg4^iii^	115.51 (14)	Ga1^v^—Mg4—Al2	94.86 (14)
Mg3—Al3—Mg4^iii^	62.87 (13)	Mg2^v^—Mg4—Al2	100.4 (2)
Mg4^ix^—Al3—Mg4^iii^	178.29 (12)		

Symmetry codes: (i) −*x*, −*y*, *z*; (ii) −*z*, *x*, *y*; (iii) *z*, *x*, *y*; (iv) −*y*, *z*, −*x*; (v) *y*, *z*, *x*; (vi) −*x*, *y*, *z*; (vii) −*z*+1/2, −*x*+1/2, −*y*+1/2; (viii) *z*−1/2, −*x*+1/2, −*y*+1/2; (ix) −*y*+1/2, −*z*+1/2, −*x*+1/2; (x) *x*, *y*, −*z*+1; (xi) −*x*+1/2, −*y*+1/2, −*z*+1/2; (xii) −*x*+1, −*y*, −*z*+1; (xiii) *y*+1/2, *z*−1/2, *x*+1/2; (xiv) −*x*+1/2, −*y*+1/2, *z*+1/2; (xv) *x*+1/2, *y*−1/2, −*z*+1/2; (xvi) *z*, −*x*, −*y*+1; (xvii) −*y*+1/2, *z*−1/2, −*x*+1/2; (xviii) −*y*+1/2, −*z*+1/2, *x*+1/2; (xix) −*y*+1/2, *z*−1/2, *x*+1/2; (xx) *x*, −*y*, *z*; (xxi) −*y*, *z*, *x*.
